# Extremely Low-Frequency Electromagnetic Field Impairs the Development of Honeybee (*Apis cerana*)

**DOI:** 10.3390/ani12182420

**Published:** 2022-09-14

**Authors:** Yingjiao Li, Chaoxia Sun, He Zhou, Hongji Huang, Yijie Chen, Xinle Duan, Shaokang Huang, Jianghong Li

**Affiliations:** 1 College of Animal Science (College of Bee Science), Fujian Agriculture and Forestry University, Fuzhou 350002, China; 2 Jinshan College of Fujian Agriculture and Forestry University, Fuzhou 350002, China; 3 Fujian Honey Bee Biology Observation Station, Ministry of Agriculture and Rural Affairs, Fuzhou 350002, China

**Keywords:** ELF-EMF, influence, *Apis cerana*, larvae, development, transcriptome

## Abstract

**Simple Summary:**

The ELF-EMF pollution generated by the increase in electrically powered devices and power lines, accompanied by economic development, has a widespread effect on surrounding organisms. Honeybees are one of the most important pollinators. The decline in the honeybee population caused by a variety factors, including EMFs, has attracted attention worldwide. It was already known that ELF-EMFs could impair the ability of learning and cognition, causing foraging bees to lose their ability to find their way home. The pollination ability of foraging bees is derived from the rearing quantity of larvae and continuous eclosion of new adult bees in the colony. However, the effect of ELF-EMFs on honeybee larvae is not clear. The aims and objectives of this study were therefore to investigate it. The results showed that ELF-EMF exposure decreases honeybee survival rate and body weight and extends the duration of development time. Transcriptome sequencing showed that ELF-EMF exposure decreases the biological process of nutrient and energy metabolism, impedes the degradation of larvae tissues and the rebuilding of pupae tissues in the metamorphosis process, and seriously interferes with the growth and development of honeybee larvae. This provides an experimental basis and new perspective for protecting honeybee populations from ELF-EMF pollution.

**Abstract:**

Increasing ELF-EMF pollution in the surrounding environment could impair the cognition and learning ability of honeybees, posing a threat to the honeybee population and its pollination ability. In a social honeybee colony, the numbers of adult bees rely on the successful large-scale rearing of larvae and continuous eclosion of new adult bees. However, no studies exist on the influence of ELF-EMFs on honeybee larvae. Therefore, we investigated the survival rate, body weight, and developmental duration of first instar larvae continuously subjected to ELF-EMF exposure. Moreover, the transcriptome of fifth instar larvae were sequenced for analyzing the difference in expressed genes. The results showed that ELF-EMF exposure decreases the survival rate and body weight of both white-eye pupae and newly emerged adults, extends the duration of development time and seriously interferes with the process of metamorphosis and pupation. The transcriptome sequencing showed that ELF-EMF exposure decreases the nutrient and energy metabolism and impedes the degradation of larvae tissues and rebuilding of pupae tissues in the metamorphosis process. The results provide an experimental basis and a new perspective for the protection of honeybee populations from ELF-EMF pollution.

## 1. Introduction

The earth itself is a huge magnet. Its geomagnetic field is composed of poles situated close to rotational poles. The magnetic field lines point upward in the southern hemisphere, run parallel to the earth’s surface at the magnetic equator and point downward in the northern hemisphere. The intensity of the geomagnetic field is highest at the two poles and lowest near the magnetic equator. It thus forms gradients running from the poles to the equator on each hemisphere [1]. The geomagnetic field provides two kinds of information: the magnetic vector provides directional information, whereas total intensity and/or inclination may provide position information. As an environmental factor, the geomagnetic field is always present everywhere and thus represents a reliable, omnipresent source of navigational information. Many animals such as fish, marine turtles, lobsters, ants and bees, monarch butterflies and birds could perceive and use the geomagnetic field for orientation and navigation [2,3,4,5,6,7,8,9]. Moreover, it has been reported that even magnetotactic bacteria and plants have also evolved to possess this magnetoreception ability [10,11,12,13,14]. Perception and response to magnetic signals is one of the mechanisms for the successful survival and reproduction of a variety of organisms.

With economic development, the generation and transmission of electric power and the usage of a variety of appliances in the home and machines in factories increased rapidly. These activities generate extra electromagnetic fields and electromagnetic radiation, becoming an omnipresent electromagnetic polluter of the environment [15]. Extremely Low-Frequency Electromagnetic Fields (below 300 Hz), produced by power transmission lines, home wiring, car electric engines, electric trains and trams and welding devices, are one of the most common electromagnetic pollutants [16]. This electromagnetic pollution affects the lives of surrounding organisms, including human beings [15,17,18,19,20].

The pollination requirement in agriculture is increasing continuously worldwide [21]. However, the population of pollinators, including honeybees, is decreasing, posing a serious threat to agricultural production and the food safety of human beings [22,23]. As a model of a social insect in scientific studies, honeybees were previously certificated with the ability to perceive and respond to the subtle alteration in magnetic fields [24,25,26]. Additionally, ELF-EMF exposure was found to reduce learning ability, alter behaviors and flight dynamics, reduce the success of foraging flights towards food sources and feeding, and induce the cognitive impairment of honeybees [27,28]. This causes numerous foraging bees to lose their ability to return home [29]. Electromagnetic fields were therefore taken as one of the factors responsible for honeybee colony collapse disorder (CCD) [30].

Besides the effect on cognition, learning ability and behaviors, ELF-EMF exposure can also induce changes in the structure of chemical compounds in the IR region corresponding to DNA, RNA, phospholipids and protein vibrations in honeybees [31], decrease the activity of aspartate aminotransferase, alanine aminotransferase, and alkaline phosphatase in honeybees’ hemolymph, cause oxidative damage, and alter the concentration of non-enzymatic antioxidants of creatinine and albumin [32,33,34]. These changes in chemical components and the variety of physiological enzyme activity caused by ELF-EMF have a serious influence on the honeybee.

As social insects, the life span of adult worker bees is around 30–50 days. Maintenance and increase in the colony size depend on the continuous eclosion of new adult bees. Any factors affecting the success of larvae development would result in an insufficient number of newly emerged adult bees and the quick decrease in the number of adult honeybees in the colony. However, the specific effect of EMF on honeybee larvae development is so far unclear. The previous report showed that EMF exposure of larva of *Drosophila melanogaster* decreases the speed of larvae climbing and their survivability, delays the development, decreases the number of offspring [35,36], and increases the number of abnormal adult flies [37]. ELF-EMF exposure could also decrease the expression level of antimicrobial peptides in larvae of *Trichoplusia ni* [38]. Therefore, we hypothesized that EMF exposure could also impair honeybee larvae development, which could further affect the honeybee population and their pollination efficiency. To determine the effect of ELF-EMF on honeybee larvae development and the larvae’s response at the gene expression level, honeybee larvae of *Apis cerana* were subjected to ELF-EMF exposure, and their survival rate, development duration, body weight, and larvae transcriptome were investigated. The result could provide a theoretical and experimental base for protecting honeybees from the pollution of ELF-EMFs.

## 2. Materials and Methods

### 2.1. Honeybee Larvae Collection and Lab Rearing

The experimental colonies of *A. cerana* were reared in the experimental apiary of the College of Animal Science (College of Bee Science), Fujian Agriculture and Forestry University. The bee larvae were obtained by the following method: three normal egg-laying queens were separately confined on empty combs by a queen excluder for laying eggs for 8 h in their original colony; Then, these queens were moved to the other side of the queen excluder in the colony so that no more eggs were laid on the combs. These eggs were then left to naturally develop and hatch. Four days later, the comb with 2-day-old larvae was swiftly transferred to the laboratory. The 2-day-old larvae were randomly transferred from the three combs into 24-well tissue culture plates with 10 µL artificial diet in each well, using the Chinese grafting tool. The artificial diet formula is detailed in the previous report [39]. The plates were kept in a dark incubator at 34 ± 1 °C, relative humidity 90 ± 2%. These larvae were moved to a new 24-well plate with fresh diet on it per day [40].

### 2.2. ELF-EMF Exposure

A total of six 24-well plates, each with twenty-four 2-day-old larvae were set up. Among them, 72 larvae in three plates that suffered no ELF-EMF exposure were taken as the control group (CK). The remaining three plates of 72 larvae were put into the ELF-EMF generator (Litian magnetic and electric Science and Technology Co., Ltd., Mianyang, China) and received ELF-EMF exposure with a field intensity of 3 mT (50 Hz) for the period from the second day of life to the end of individual development, which was taken as the ELF-EMF exposure group (EMF). These larvae were reared as stated above. A handheld magnetic field detector (HT201, Zhongye Jingke Instrument and Equipment Co., Ltd., Guangdong, China) was used for guaranteeing the larvae in the CK group suffered no ELF-EMF exposure and the larvae in EMF group suffered the right intensity of ELF-EMF exposure. Dead larvae without worming activity inspected under the microscope were removed and recorded every day for calculating the survival rate. After defecation, these larvae were moved to new 24-well plates with three layers of sterilized filter paper in the well for their pupation, and then kept in the incubator at 34 °C, 75% RH for the pupae stage. Both the white eye pupae and the newly emerged adults were weighted for determining the effect of ELF-EMF on honeybee weight. Meanwhile, the emerging time of each pupa was recorded for determining the effect of ELF-EMF on the duration of honeybee development.

### 2.3. RNA Extraction, Library Construction, Sequencing, Reads Mapping and Assembling

Food and nutrients fundamentally determined the development of honeybee larvae. Feeding amount and its utilization by the larvae to a great extent determined the weight and development of the honeybee. The fifth instar larvae are the maximum feeding stage of the larvae. Thereby, three fifth-instar larvae from the CK group and EMF group, respectively, were randomly collected for RNA extraction and the following transcriptome sequencing and bioinformatic analysis for investigating the response to ELF-EMF at the gene expression level. In detail, total RNA was extracted from these fifth instar larvae using TRIzol^®^ reagent (Invitrogen, Carlsbad, CA, USA), and RNA integrity was assessed using the RNA Nano 6000 Assay Kit of the Bioanalyzer 2100 system (Agilent Technologies, Palo Alto, CA, USA). Sequencing libraries were generated using the NEBNext^®^ Ultra™ RNA Library Prep Kit for Illumina^®^ (New England Biolabs, Ipswich, MA, USA) according to the manufacturer’s recommendations. The libraries were sequenced on an Illumina Novaseq platform and 150 bp paired-end reads were generated. The sequenced raw reads were further processed for obtaining the clean data (clean reads) by removing reads containing adapter, reads containing ploy-N and low-quality reads. Finally, a total of 37.16 Gb clean reads were obtained. Reference genome and gene model annotation files were downloaded from the genome website directly (https://ftp.ncbi.nlm.nih.gov/genomes/all/GCF/001/442/555/GCF_001442555.1_ACSNU-2.0/GCF_001442555.1_ACSNU-2.0_genomic.gff.gz, accessed on 17 December 2019). Index of the reference genome was built using Hisat2 and paired-end clean reads were aligned to the reference genome using Hisat2 [41]. The mapped reads of each sample were assembled by StringTie [42] in a reference-based approach.

### 2.4. Quantification and Differential Gene Expression Analysis

The expression levels of each unigene were analyzed using the RPKM method (reads per kilobase of transcript per million reads mapped). FeatureCounts v1.5.0 [43] was used to count the read numbers mapped to each gene. Additionally, FPKM of each gene was calculated based on the length of the gene and the read count mapped to this gene. Differential expression analysis between the two groups of honeybee larvae was performed using the DESeq2 R package [44]. The resulting *p*-values were adjusted using Benjamini and Hochberg’s approach for controlling the false discovery rate. Genes with an adjusted *p*-value < 0.05 found by DESeq2 were assigned as differentially expressed.

### 2.5. GO and KEGG Enrichment Analysis of Differentially Expressed Genes

Gene Ontology (GO) enrichment and KEGG pathway analysis of all differentially expressed genes were implemented by the cluster Profiler R package [45], in which gene length bias was corrected. GO terms with a corrected *p*-value less than 0.05 were considered significantly enriched by differentially expressed genes.

### 2.6. Validation of the Gene Expression Level by qRT-PCR

To validate the reliability of the transcriptome sequencing data, the expressions of six genes from the KEGG enrichment (*elovel 1*, *elovel 4*, *elovel 6*, *fas*, *vg*, and *chitinase*) related to the energy production and nutrients uptake and utilization were quantified by qRT-PCR (SYBR Green Master Mix, Yeasen Biotech Co., Ltd., Shanghai, China) using the designed specific primers (Table S1). The reactions were performed as follows: 95 °C for 3 min, followed by 45 cycles of denaturation at 95 °C for 15 s, then annealing at 60 °C for 20 s, and extension at 72 °C for 20 s. The *β-actin* was used as a reference [46]. All of the reactions were performed with three biological replicates. The reactions were performed with an ABI QuantStudio 6 Flex System (Thermo Fisher Scientific, Waltham, MA, USA).

### 2.7. Statistical Analysis

All experimental data were expressed as the mean ± SD (standard deviation). Significant differences between the ELF-EMF exposure group and the control group were assessed by Duncan’s post hoc test and Dunnett’s *t*-test with a confidence level of 95%. The survival curve was constructed based on the data of daily dead larvae number collected, and the difference between the two test groups was analyzed using the Log-rank (Mantel-Cox) test in GraphPad Prism (Version 7.0, Graph Pad Software Inc., San Diego, CA, USA). The threshold cycle (CT) obtained from the qRT-PCR was determined and the 2^−ΔΔCt^ equation was used to calculate the gene expression levels. The difference between the expression level from the qRT-PCR and transcriptome sequencing was analyzed by *t*-test in GraphPad Prism 7. The significance level was set at a value of *p* < 0.05.

## 3. Results

### 3.1. ELF-EMF Exposure Decreased the Larvae Survival Rate

**Figure 1:**
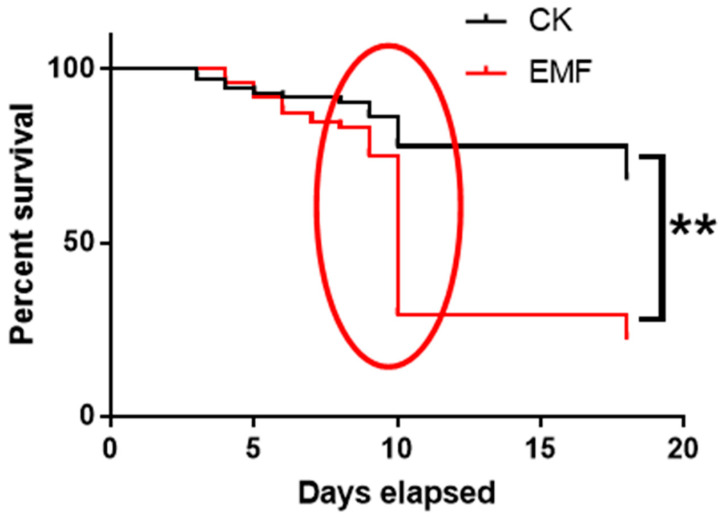
Effect of ELF-EMF exposure on the survival of *A. cerana* larvae. EMF: ELF-EMF exposed group (Red line). CK: Control group (Black line); Survival curves show ELF-EMF exposure significantly decreased the survivorship of honeybee larvae (Log-rank test, *χ*^2^ = 28.24, *p* < 0.001), especially for the larvae in the process of metamorphosis and pupation (red line circled part). Note: ** means *p* < 0.01.

The survival curve of honeybee larvae was prepared using the daily dead honeybee number. The result showed that 68% of larvae in the control group could finish their development, which was only 22% in the EMF exposure group. ELF-EMF exposure significantly decreased the survival rate of honeybee larvae (Log-rank test, χ^2^ = 28.24, *p* < 0.001). Most larvae from the EMF group died at the ninth and tenth day corresponding to the metamorphosis stage (Figure 1). ELF-EMF seriously interfered with the metamorphosis and pupation process of honeybee larvae.

### 3.2. ELF-EMF Exposure Decreased the Honeybee Weight

**Figure 2:**
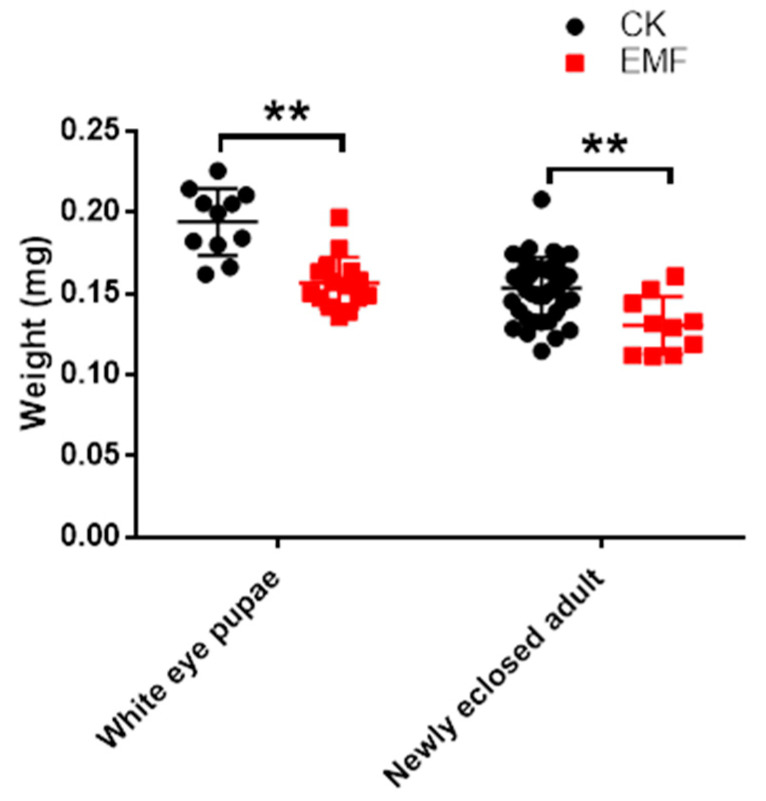
Effect of ELF-EMF exposure on the weight of while-eye pupae and newly emerged adults of *A. cerana*. EMF: ELF-EMF exposed group (Red square). CK: Control group (Black dot); ELF-EMF exposure significantly decreased the weight of white-eye pupae and newly emerged adult (*t*-test: for the white eye pupae, *t* = 6.623, *df* = 22, *p* < 0.0001; for the newly emerged adult, *t* = 3.427, *df* = 47, *p* = 0.0013). Note: ** means *p* < 0.01.

By investigating the weight of white eye pupae and newly emerged adults, respectively, we found that both the weight of white eye pupae (0.1518 ± 0.0027 g, n = 13) and newly emerged adults (0.1305 ± 0.0056 g, n = 10) from the ELF-EMF exposure group were significantly lower than the weight of white eye pupae (0.1942 ± 0.0062 g, n = 11) and newly emerged adults (0.1533 ± 0.0031 g, n = 39) from the control group (Independent *t*-test: for the white eye pupae, *t* = 6.623, *df* = 22, *p* < 0.0001; for the newly emerged adult, *t* = 3.427, *df* = 47, *p* = 0.0013) (Figure 2). Thereby, ELF-EMF exposure significantly decreased both the weight of white eye pupae and newly emerged adults.

### 3.3. ELF-EMF Exposure Extended the Honeybee Development Time

**Figure 3:**
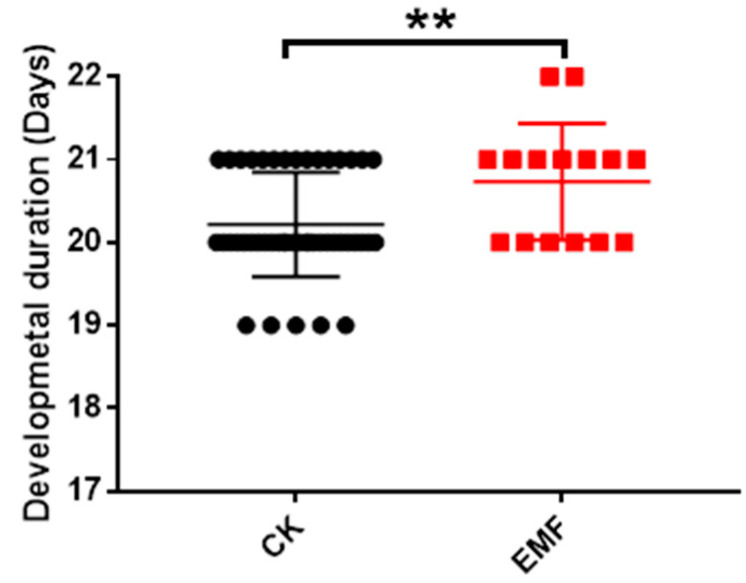
Effect of ELF-EMF exposure on the development duration of honeybee larvae. EMF: ELF-EMF exposed group (Red square). CK: Control group (Black dot); ELF-EMF exposure significantly increased the developmental time of honeybee larvae (*t*-test, *t* = 2.747, *df* = 61, *p* = 0.0079). Note: ** means *p* < 0.01.

By analyzing the emerging time of these test pupae, the result showed that the total developmental time of larvae from the EMF group was 20.73 ± 0.1817 days (n = 15), which was significantly longer than the developmental time (20.13 ± 0.1099 days, n = 48) of larvae from the control group (Independent *t*-test, *t* = 2.747 *df* = 61, *p* = 0.0079) (Figure 3). Thereby, ELF-EMF exposure significantly increased the developmental time of honeybee larvae.

### 3.4. Transcriptome Sequencing

Six libraries of the fifth instar larvae from the control groups and ELF-EMF exposure groups were constructed and sequenced. The number of clean reads obtained for each library ranged from 38,475,672 to 45,990,156, with data produced ranging from 5.77 G to 6.90 G. The average value of Q30 was 93.455. The clean reads of all samples had a good mapping rate to the genome of *A. cerana* ranging from 87.33% to 89.70%. This showed that the transcriptome libraries were constructed successfully. A total of 1037 novel transcripts were assembled using the software StringTie. Together with these mapped unigenes, a total of 10,972 unigenes were obtained and used for further analysis. The transcriptome sequence data of the six libraries were deposited in the Sequence Read Archives (SRA) at NCBI under accession numbers (SRS10030307, SRS10030308, SRS10030309, SRS10030310, SRS10030311, and SRS10030312) under BioProject ID: PRJNA760941.

### 3.5. Differentially Expressed Genes Analysis

**Figure 4:**
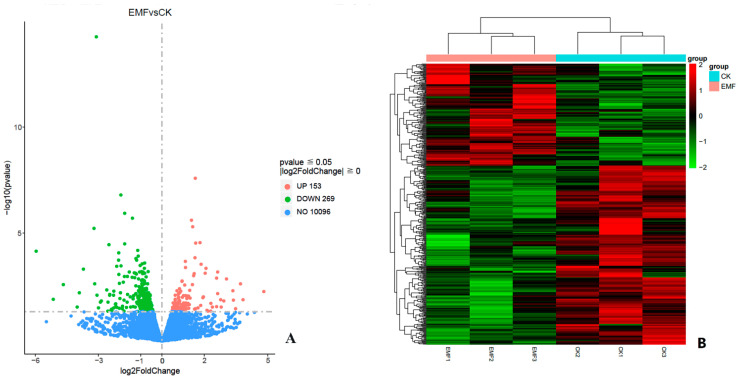
Volcano plot (**A**) and hierarchical clustering (**B**) of differentially expressed unigenes (DEGs) in honeybee larvae from EMF groups and CK groups. The expression level for each unigene was shown in the volcano plot. Up, up-regulated; Down, downregulated. *X*-axis shows fold change of the gene between EMF and CK groups. *Y*-axis means Log10 of unigene. The significant DEGs were considered as fold change ≥ 1 and FDR ≤ 0.05. Clustering analysis of DEGs showed that their expression mode in the three control samples could be clustered in one branch, while that in the three ELF-EMF exposed samples could be clustered in another branch. The similar expression profiles of DEGs in the three samples of both EMF group and CK group implied the good repeatability of the test.

Differentially expressed genes (DEGs) in honeybee larvae between the CK group and EMF group were analyzed. Among the 10,972 unigenes, a total of 422 unigenes showed different expressions, including 153 up-regulated unigenes and 269 downregulated unigenes in group EMF (Figure 4A). Clustering analysis showed that the expression mode of these DEGs in the three samples from the CK group could be clustered in one branch, while those in the three samples from the EMF group could be clustered in another branch (Figure 4B). The similar expression profiles of DEGs in the three samples of both the EMF group and CK group implied the good repeatability of the three prepared samples and the follow sequencing.

### 3.6. GO Enrichment

**Figure 5:**
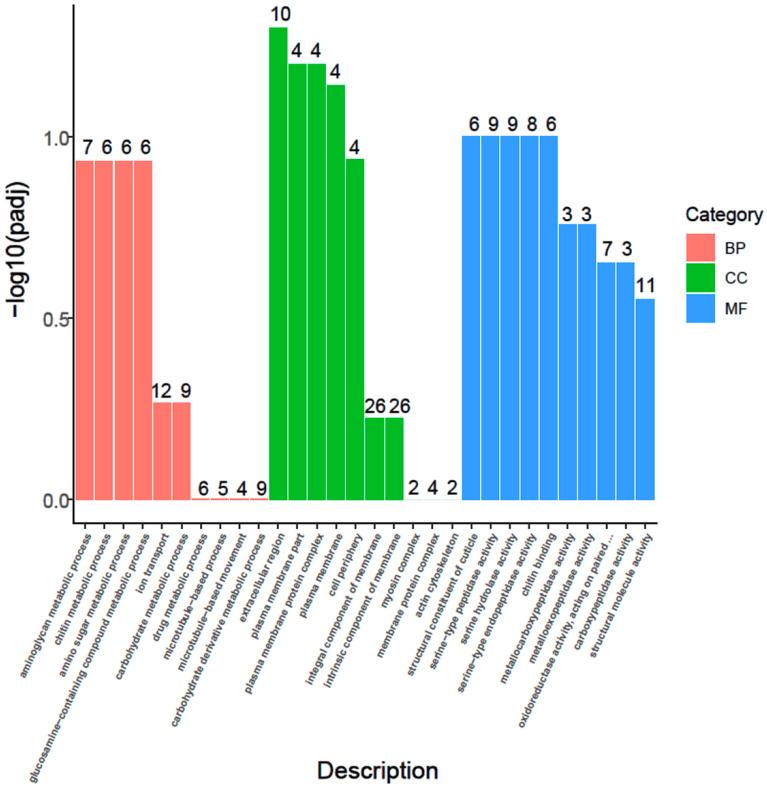
GO enrichment result of the differentially expressed unigenes (DEGs) in honeybee larvae from the EMF group and CK group. GO enrichment result showed that the ELF-EMF exposure mainly interfere the structure and function of plasma membrane, the structural constituent and function of cuticle, and variety of peptidase activity.

GO annotation analyses were performed to identify the function of DEGs. Based on GO annotation, the 422 DEGs were classified into three major categories: biological process (114 DEGs), cellular component (73 DEGs), and molecular function (191 DEGs). The most enriched GO items in the biological process were the aminoglycan metabolic process, chitin metabolic process, amino sugar metabolic process, glucosamine-containing compound metabolic process, ion transport and carbohydrate metabolic process. The most enriched GO items in the cellular component were the extracellular region, plasma membrane part, plasma membrane protein complex, plasma membrane, cell periphery integral component of membrane and intrinsic component of membrane. Additionally, the most enriched GO items in the molecular function were the structural constituent of cuticle, serine-type peptidase activity, serine hydrolase activity, serine-type endopeptidase activity, chitin binding, metallocarboxypeptidase activity, metalloexopeptidase activity, oxidoreductase activity, acting on paired donors, with incorporation or reduction in molecular oxygen, carboxypeptidase activity and structural molecule activity (Figure 5). Thereby, these DEGs caused by ELF-EMF exposure were mainly involved in the structure and function of plasma membrane, the structural constituent and function of cuticle, and variety of peptidase activity.

### 3.7. KEGG Enrichment

**Figure 6:**
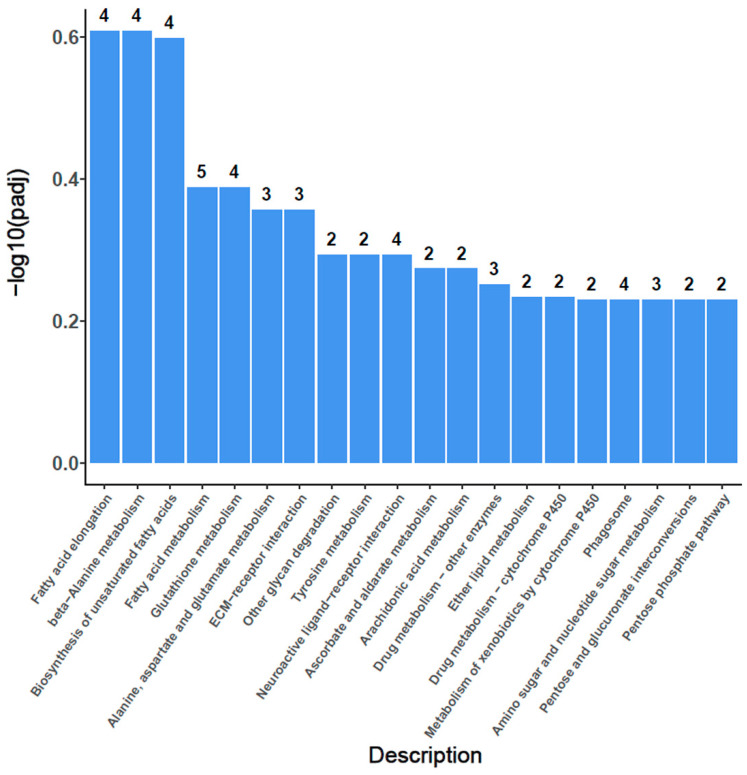
KEGG pathways of the differentially expressed unigenes in honeybee larvae from the EMF group and CK group. KEGG pathways enrichment showed that ELF-EMF exposure mainly decreased the activity of nutrient and energy metabolism honeybee larvae.

KEGG enrichment analyses were performed to identify the pathways of DEGs. A total of twelve pathways were enriched. Among them, only two pathways of glycan degradation and beta-Alanine metabolism were unregulated. The other ten pathways were all downregulated. They were fatty acid elongation, biosynthesis of unsaturated fatty acids, fatty acid metabolism, tyrosine metabolism, drug metabolism or other enzymes, glutathione metabolism, ascorbate and aldarate metabolism, arachidonic acid metabolism, phagosome, and ether lipid metabolism. All these downregulated pathways are mainly involved in the metabolism of carbohydrates, fatty acids and amino acids, which was related to the process of energy production and nutrient uptake and utilization (Figure 6). Therefore, ELF-EMF exposure mainly decreased the activity of nutrient and energy metabolism honeybee larvae.

### 3.8. Validation by qRT-PCR

**Figure 7:**
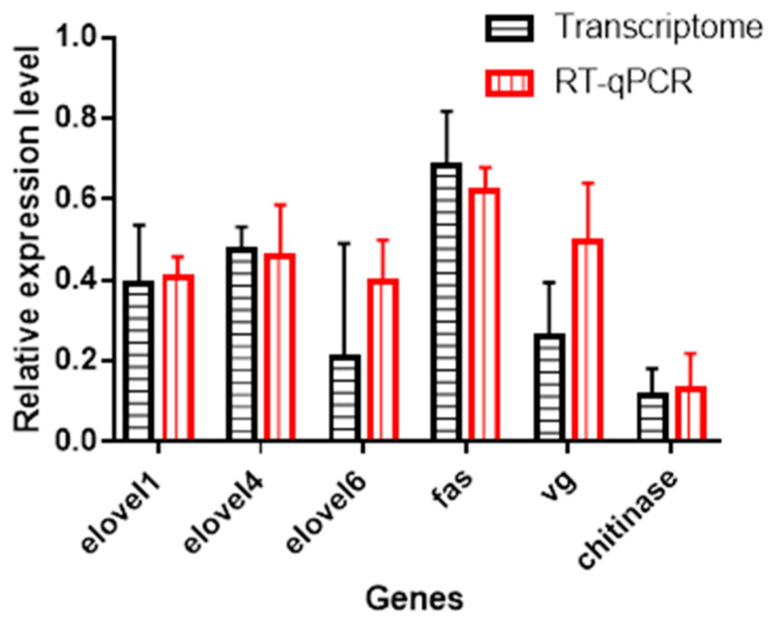
qRT-PCR validation of the partial differentially expressed unigenes (DEGs) in honeybee larvae. qRT-PCR: Relative expression of six selected genes by qRT-PCR (Red column). Transcriptome: Relative expression of six selected genes by transcriptome analysis (Black column); The expression of six genes selected from the KEGG enrichment in the larvae from the EMF group were all downregulated when compared with the expression in the larvae from the CK group by the method of qRT–PCR, which was identical to their corresponding expression level shown in RNA-seq analysis (*t*-test, for all the six genes, *p* > 0.05). This confirmed the accuracy of the RNA-seq results.

Six enriched genes from the KEGG pathway (*elovel 1*, *elovel 4*, *elovel 6*, *fas*, *vg* and *chitinase*) related to energy production and nutrients uptake and utilization were quantified by qRT-PCR using the designed specific primers (Table S1). The dissolution curve of all of the primers had a single peak without any miscellaneous peak. The relative mRNA expression of all the six genes in larvae from EMF group were about 15–70% of the expression level in larvae from CK group assessed by qRT–PCR. Such a downregulated expression profile in qRT-PCR was identical to their expression mode shown in the RNA-seq analysis (*t*-test, for all the six genes, *p* > 0.05) (Figure 7). This confirmed the accuracy of the RNA-seq results.

## 4. Discussion

The honeybee population, especially the number of foraging adult bees in the colony, is the determiner of its pollination ability. Previous reports showed that the ELF- EMF could impair honeybee cognition and reduce the success of foraging flights [27,28], causing the decrease in foraging bees, and threatening their ability to meet the pollination requirement of crops. However, the numbers of adult bees depend on the incessant eclosion of new adults from the large number of larvae bred in the colony. Any effect on larvae breeding or development would result in the immediate decrease in adult bees. ELF-EMF is one of the most common environmental pollutants affecting living organisms. However, its effect on larvae development is not clear. Our results showed that ELF-EMF exposure decreased the body weight and survival rate of honeybee larvae and extended the duration of their development period. Such results were similar to the influence of ELF-EMF on the larvae of *Drosophila melanogaster* [36,37], verifying that ELF-EMF could also decrease the honeybee population by affecting larvae development and survival rate. This warns us of the negative effect of ELF-EMF exposure on honeybee larvae, and simultaneously provides a new perspective to protect the honeybee population from ELF-EMF exposure. It is extremely necessary to consider the electromagnetic pollution of the environment in beekeeping and keep the apiary construction or colonies placement far away from the site of generation and transmission of electric power and factories using a variety of electric equipment.

The honeybee is a complete metamorphosis insect. The metamorphosis involved the degradation of the larvae tissues and reconstruction of the pupae tissues. Any interference or disturbance of the metamorphosis process would cause the larvae pupation to fail. The survival rate showed that ELF-EMF exposure caused many larvae dead, especially in the pupation process (Figure 1). Such results showed that ELF-EMF exposure seriously interfered with the metamorphosis and pupation process of honeybee larvae.

Seeing that ELF-EMF exposure decreases honeybee body weight and extends the duration of their development period, the interference of nutrient synthesis and metabolism should be involved in larvae caused by ELF-EMF exposure. The fifth-instar larvae is the stage of maximum feeding. Therefore, the 5th instar ELF-EMF-exposed larvae were subjected to transcriptome sequencing for analyzing the differently expressed genes and their enriched GO and KEGG pathway. GO analysis of the differently expressed genes showed that the structure and function of the plasma membrane, the structural constituent and function of the cuticle, and the variety of peptidase activity were mostly enriched (Figure 5). The honeybee larvae absorb and store nutrient substances in body fat cells. These body fat cells will break up in the pupation process and the stored nutrient substance will be degraded and released to the hemolymph for constructing the tissue of pupae [47].This process involves the active degradation of the stored protein (corresponding to the peptidase activity in GO enrichment) and disruption of the larvae plasma membrane of body fat cells and the formation of the plasma membrane of pupae tissues (corresponding to the structure and function of the plasma membrane in GO enrichment). The pupation process also involved larvae molting and pupae cuticle formation (corresponding to the structural constituent and function of cuticle in GO enrichment). Therefore, GO enrichment simultaneously showed that ELF-EMF exposure could deeply interfere with the pupation process of the honeybee, causing numerous larvae to die in the process (Figure 1).

KEGG pathway enrichment of the differentially expressed genes caused by ELF-EMF exposure showed that most of the enriched pathways are downregulated. They are mainly involved in the synthesis and metabolism of fatty acids and the metabolism of amino acids and carbohydrates (Figure 6). Such results showed that ELF-EMF exposure downregulated the synthesis and metabolism of nutrients and energy metabolism. The newly-hatched honeybee larvae with a body weight of around 0.1 mg grow quickly to nearly 200 mg within 6 days of the larvae stage [48]. This cannot occur without the active metabolism of nutrients and energy. ELF-EMF exposure downregulated the metabolism of nutrients and energy, which seriously interfered with the normal growth and development of honeybee larvae and caused a decrease in body weight (Figure 2). Moreover, the normal pupation process is also an active physiological process involving the active degradation of larvae tissues and rebuilding of pupae tissues, which also require plenty of energy supply. The deficiency in nutrient and energy supply might also lead to the failure of pupation. That might be the main reason for the failed pupation of honeybee larvae in the ELF-EMF exposure groups (Figure 1).

## 5. Conclusions

Increasing ELF-EMF environmental pollution threatens the survival of foraging honeybees. Healthy larvae and the continuous eclosion of new adult bees in the colony fundamentally determine the number of adult bees and their pollination efficiency. Therefore, the effect of ELF-EMF on honeybee larvae, both from the biology level and background molecular level, deserve to be determined. Our results showed that ELF-EMF exposure decreased the survival rate and body weight of honeybees, extended the duration of their development time, and seriously interfered with the process of metamorphosis and pupation. GO enrichment of the differentially expressed genes showed that the structure and function of the plasma membrane, the structural constituent and function of cuticle, and variety of peptidase activity were mostly enriched. KEGG enrichment showed that these pathways of the synthesis and metabolism of fatty acids and the metabolism of amino acids and carbohydrates were mostly enriched. ELF-EMF exposure decreases nutrient and energy metabolism and interferes with the process of metamorphosis and pupation. The results verified the negative effect of ELF-EMF on the growth and development of honeybee larvae and provide us with a new perspective on the protection of the honeybee populations from ELF-EMF pollution.

## Data Availability

The sequence data of transcriptome were deposited in the Sequence Read Archives (SRA) at NCBI under accession numbers (SRS10030307, SRS10030308, SRS10030309, SRS10030310, SRS10030311, and SRS10030312) under BioProject ID: PRJNA760941. The other data sets generated and/or analyzed during the current study are available from the corresponding author on reasonable request.

## Supplementary Materials

The following supporting information can be downloaded at: https://www.mdpi.com/article/10.3390/ani12182420/s1, Figure S1: the photo of honeybee larvae subjecting to the ELF-EMF exposure; Table S1: primers used for validation of the level of gene expression in qRT-PCR; Table S2: sequencing quality of six Transcriptome library; Table S3: mapping rate of the sequencing reads to the reference genome of *A. cerana*.

## References

[1] Skiles D.D., Kirschvink J.L., Jones D.S., MacFadden B.J. (1985). The Geomagnetic Field Its Nature, History, and Biological Relevance. Magnetite Biomineralization and Magnetoreception in Organisms: A New Biomagnetism.

[2] Formicki K., Korzelecka-Orkisz A., Tański A. (2019). Magnetoreception in fish. J. Fish Biol..

[3] Naisbett-Jones L.C., Lohmann K.J. (2022). Magnetoreception and magnetic navigation in fishes: A half century of discovery. J. Comp. Physiol. A.

[4] Lohmann K., Lohmann C. (1996). Orientation and open-sea navigation in sea turtles. J. Exp. Biol..

[5] Wiltschko W., Wiltschko R. (2005). Magnetic orientation and magnetoreception in birds and other animals. J. Comp. Physiol. A.

[6] Fleischmann P.N., Grob R., Rössler W. (2020). Magnetoreception in Hymenoptera: Importance for navigation. Anim. Cogn..

[7] Reppert S.M., Gegear R.J., Merlin C. (2010). Navigational mechanisms of migrating monarch butterflies. Trends Neurosci..

[8] Mouritsen H., Ritz T. (2005). Magnetoreception and its use in bird navigation. Curr. Opin. Neurobiol..

[9] Wiltschko R., Wiltschko W. (2019). Magnetoreception in birds. J. R. Soc. Interface.

[10] Goswami P., He K., Li J., Pan Y., Roberts A.P., Lin W. (2022). Magnetotactic bacteria and magnetofossils: Ecology, evolution and environmental implications. NPJ Biofilms Microbiomes.

[11] Lin W., Kirschvink J.L., Paterson G.A., Bazylinski D.A., Pan Y. (2020). On the origin of microbial magnetoreception. Natl. Sci. Rev..

[12] Monteil C.L., Lefevre C.T. (2020). Magnetoreception in Microorganisms. Trends Microbiol..

[13] Jacob J.J., Suthindhiran K. (2016). Magnetotactic bacteria and magnetosomes—Scope and challenges. Mater. Sci. Eng. C.

[14] Maffei M.E. (2014). Magnetic field effects on plant growth, development, and evolution. Front. Plant Sci..

[15] Redlarski G., Lewczuk B., Żak A., Koncicki A., Krawczuk M., Piechocki J., Jakubiuk K., Tojza P., Jaworski J., Ambroziak D. (2015). The influence of electromagnetic pollution on living organisms: Historical trends and forecasting changes. Biomed. Res. Int..

[16] Ahlbom A., Bridges J., de Seze R., Hillert L., Juutilainen J., Mattsson M.O., Neubauer G., Schüz J., Simko M., Bromen K. (2008). Possible effects of electromagnetic fields (EMF) on human health—Opinion of the scientific committee on emerging and newly identified health risks (SCENIHR). Toxicology.

[17] Wyszkowska J., Shepherd S., Sharkh S., Jackson C.W., Newland P.L. (2016). Exposure to extremely low frequency electromagnetic fields alters the behaviour, physiology and stress protein levels of desert locusts. Sci. Rep..

[18] Jadidi M., Firoozabadi S.M., Rashidy-Pour A., Sajadi A.A., Sadeghi H., Taherian A.A. (2007). Acute exposure to a 50Hz magnetic field impairs consolidation of spatial memory in rats. Neurobiol. Learn. Mem..

[19] Szemerszky R., Zelena D., Barna I., Bárdos G. (2010). Stress-related endocrinological and psychopathological effects of short- and long-term 50 Hz electromagnetic field exposure in rats. Brain Res. Bull..

[20] Repacholi M. (2012). Concern that “EMF” magnetic fields from power lines cause cancer. Sci. Total Environ..

[21] Breeze T.D., Vaissière B.E., Bommarco R., Petanidou T., Seraphides N., Kozák L., Scheper J., Biesmeijer J.C., Kleijn D., Gyldenkærne S. (2014). Agricultural policies exacerbate honeybee pollination service supply-demand mismatches across Europe. PLoS ONE.

[22] Potts S.G., Biesmeijer J.C., Kremen C., Neumann P., Schweiger O., Kunin W.E. (2010). Global pollinator declines: Trends, impacts and drivers. Trends Ecol. Evol..

[23] Gallai N., Salles J.-M., Settele J., Vaissière B.E. (2009). Economic valuation of the vulnerability of world agriculture confronted with pollinator decline. Ecol. Econ..

[24] Kirschvink J., Padmanabha S., Boyce C., Oglesby J. (1997). Measurement of the threshold sensitivity of honeybees to weak, extremely low-frequency magnetic fields. J. Exp. Biol..

[25] Walker M.M., Bitterman M.E. (1989). Conditioning analysis of magnetoreception in honeybees. Bioelectromagnetics.

[26] Hsu C.Y., Li C.W. (1994). Magnetoreception in honeybees. Science.

[27] Shepherd S., Lima M.A.P., Oliveira E.E., Sharkh S.M., Jackson C.W., Newland P.L. (2018). Extremely Low Frequency Electromagnetic Fields impair the Cognitive and Motor Abilities of Honey Bees. Sci. Rep..

[28] Shepherd S., Hollands G., Godley V.C., Sharkh S.M., Jackson C.W., Newland P.L. (2019). Increased aggression and reduced aversive learning in honey bees exposed to extremely low frequency electromagnetic fields. PLoS ONE.

[29] Migdał P., Berbeć E., Bieńkowski P., Plotnik M., Murawska A., Latarowski K. (2022). Exposure to Magnetic Fields Changes the Behavioral Pattern in Honeybees (*Apis mellifera* L.) under Laboratory Conditions. Animals.

[30] Santhosh Kumar S. (2018). Colony Collapse Disorder (CCD) in Honey BeesCaused by EMF Radiation. Bioinformation.

[31] Koziorowska A., Depciuch J., Białek J., Woś I., Kozioł K., Sadło S., Piechowicz B. (2020). Electromagnetic field of extremely low frequency has an impact on selected chemical components of the honeybee. J. Comp. Physiol. A Neuroethol. Sens. Neural Behav. Physiol..

[32] Migdał P., Murawska A., Bieńkowski P., Strachecka A., Roman A. (2021). Effect of the electric field at 50 Hz and variable intensities on biochemical markers in the honey bee’s hemolymph. PLoS ONE.

[33] Duan Y., Wang Z., Zhang H., He Y., Lu R., Zhang R., Sun G., Sun X. (2013). The preventive effect of lotus seedpod procyanidins on cognitive impairment and oxidative damage induced by extremely low frequency electromagnetic field exposure. Food Funct..

[34] Migdał P., Murawska A., Strachecka A., Bieńkowski P., Roman A. (2020). Changes in the Honeybee Antioxidant System after 12 h of Exposure to Electromagnetic Field Frequency of 50 Hz and Variable Intensity. Insects.

[35] Atli E., Unlü H. (2006). The effects of microwave frequency electromagnetic fields on the development of *Drosophila melanogaster*. Int. J. Radiat. Biol..

[36] Agrawal N., Verma K., Baghel D., Chauhan A., Prasad D.N., Sharma S.K., Kohli E. (2021). Effects of extremely low-frequency electromagnetic field on different developmental stages of *Drosophila melanogaster*. Int. J. Radiat. Biol..

[37] Mirabolghasemi G., Azarnia M. (2002). Developmental changes in *Drosophila melanogaster* following exposure to alternating electromagnetic fields. Bioelectromagnetics.

[38] Valadez-Lira J.A., Medina-Chavez N.O., Orozco-Flores A.A., Heredia-Rojas J.A., Rodriguez-de la Fuente A.O., Gomez-Flores R., Alcocer-Gonzalez J.M., Tamez-Guerra P. (2017). Alterations of Immune Parameters on *Trichoplusia ni* (Lepidoptera: Noctuidae) Larvae Exposed to Extremely Low-Frequency Electromagnetic Fields. Environ. Entomol..

[39] Vojvodic S., Jensen A.B., James R.R., Boomsma J.J., Eilenberg J. (2011). Temperature dependent virulence of obligate and facultative fungal pathogens of honeybee brood. Vet. Microbiol..

[40] Jensen A.B., Pedersen B.V., Eilenberg J. (2009). Differential susceptibility across honey bee colonies in larval chalkbrood resistance. Apidologie.

[41] Mortazavi A., Williams B.A., McCue K., Schaeffer L., Wold B. (2008). Mapping and quantifying mammalian transcriptomes by RNA-Seq. Nat. Methods.

[42] Pertea M., Pertea G.M., Antonescu C.M., Chang T.-C., Mendell J.T., Salzberg S.L. (2015). StringTie enables improved reconstruction of a transcriptome from RNA-seq reads. Nature Biotechnol..

[43] Liao Y., Smyth G.K., Shi W. (2013). Feature counts: An efficient general purpose program for assigning sequence reads to genomic features. Bioinformatics.

[44] Love M.I., Huber W., Anders S. (2014). Moderated estimation of fold change and dispersion for RNA-seq data with DESeq2. Genome Biol..

[45] Yu G., Wang L.G., Han Y., He Q.Y. (2012). Cluster profiler: An R package for comparing biological themes among gene clusters. Omics A J. Integr. Biol..

[46] Simone M., Evans J.D., Spivak M. (2009). Resin collection and social immunity in honey bees. Evol. Int. J. Org. Evol..

[47] Telfer W.H., Kunkel J.G. (1991). The function and evolution of insect storage hexamers. Annu. Rev. Entomol..

[48] Zoltowska K., Frączek R., Lipiński Z. (2011). Hydrolases of developing worker brood and newly emerged worker of *Apis mellifera carnica*. J. Apic. Sci..

