# Supplementary figures and images for: Extremely Low-Frequency Electromagnetic Field Impairs the Development of Honeybee (Apis cerana)
Source: Animals (Basel). 2022 Sep 14;12(18):2420. doi: 10.3390/ani12182420 (PMC9495099; doi:10.3390/ani12182420)

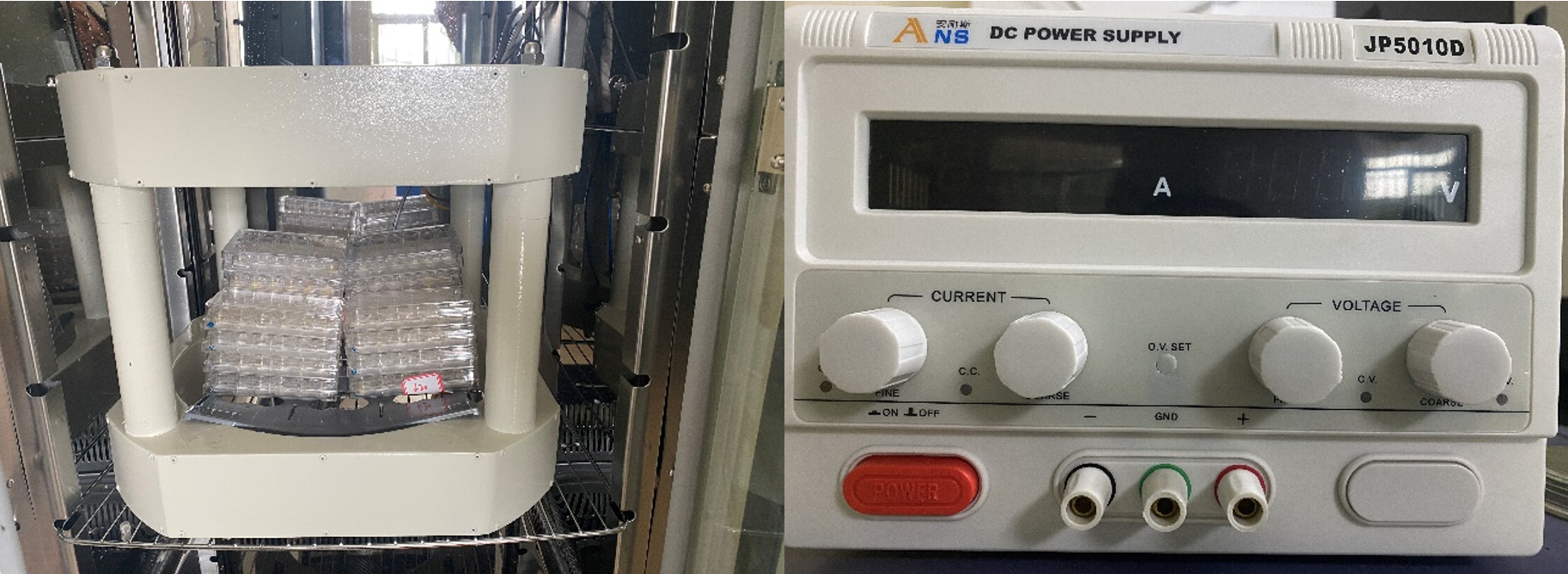

Supplement: Supplementary file 1 [file animals-12-02420-s001.zip › animals-1894676-supplementary/Figrue S1.jpg]
